# Molecular and developmental insights into proprioceptive identity in the mesencephalic trigeminal nucleus neurons

**DOI:** 10.3389/fncel.2025.1716643

**Published:** 2026-03-09

**Authors:** Pa Reum Lee, Seog Bae Oh

**Affiliations:** 1 Brain Science Institute, Korea Institute of Science and Technology, Seoul, Republic of Korea; 2 Department of Neurobiology and Physiology, School of Dentistry and Dental Research Institute, Seoul National University, Seoul, Republic of Korea; 3 ADA Forsyth Institute, Somerville, MA, United States

**Keywords:** developmental origins, dorsal root ganglion, mastication, mesencephalic trigeminal nucleus, oral sensorimotor control, postnatal development, proprioceptive primary sensory neuron, transcriptional programs

## Abstract

The mesencephalic trigeminal nucleus (MTN) contains trigeminal proprioceptive neurons, a unique class of primary sensory neurons with centrally located cell bodies and a developmental origin distinct from that of peripheral ganglion-derived spinal proprioceptors. MTN neurons have long been recognized for their morphological heterogeneity, but their functions were traditionally viewed as confined to the jaw jerk reflex and oromotor control, reflecting their predominant innervation of jaw-closing muscles. Recent single-cell transcriptomic studies have provided new insight into MTN neurons by uncovering molecular determinants of proprioceptive identity, revealing discrete transcriptional programs that underlie their developmental trajectories and functional specialization. While some subsets of MTN neurons share features with Group Ia and II proprioceptors, they are distinguished by characteristic molecular signatures, including the absence of *Runx3*, differential *Ntrk2* and *Ntrk3* expression, and broader transcriptional features that are not observed in classical spinal counterparts. Accumulating evidence also supports a functional role for MTN neurons in the behavioral transition from suckling to mastication during the weaning period in mammals. In this review, we integrate anatomical, molecular, and functional perspectives to refine the proprioceptive identity of MTN neurons and highlight their implications for sensorimotor maturation and developmental disorders.

## 1 Introduction

The mesencephalic trigeminal nucleus (MTN), also referred to as Mes V (mesencephalic nucleus of the fifth nerve), contains first-order cranial sensory neurons whose cell bodies reside in the brainstem, representing an exception to the canonical organization of sensory neurons in peripheral ganglia (Corbin, 1940; Jerge, 1963). Early anatomical studies, building on late nineteenth-century descriptions by Meynert and including the comparative analyses of Johnston (1909) and the detailed morphological descriptions by Ramón y Cajal (1909), revealed the atypical localization of the MTN within the central nervous system, thereby challenging the classical boundary between the peripheral and central nervous systems (Alvarado-Mallart et al., 1975; Vermeiren et al., 2020). MTN neurons are recognized as a unique class of proprioceptors within the trigeminal sensory system, yet they arise from a developmental lineage distinct from that of limb-innervating proprioceptors of the dorsal root ganglia (DRG) (Vermeiren et al., 2020).

**Figure 1:**
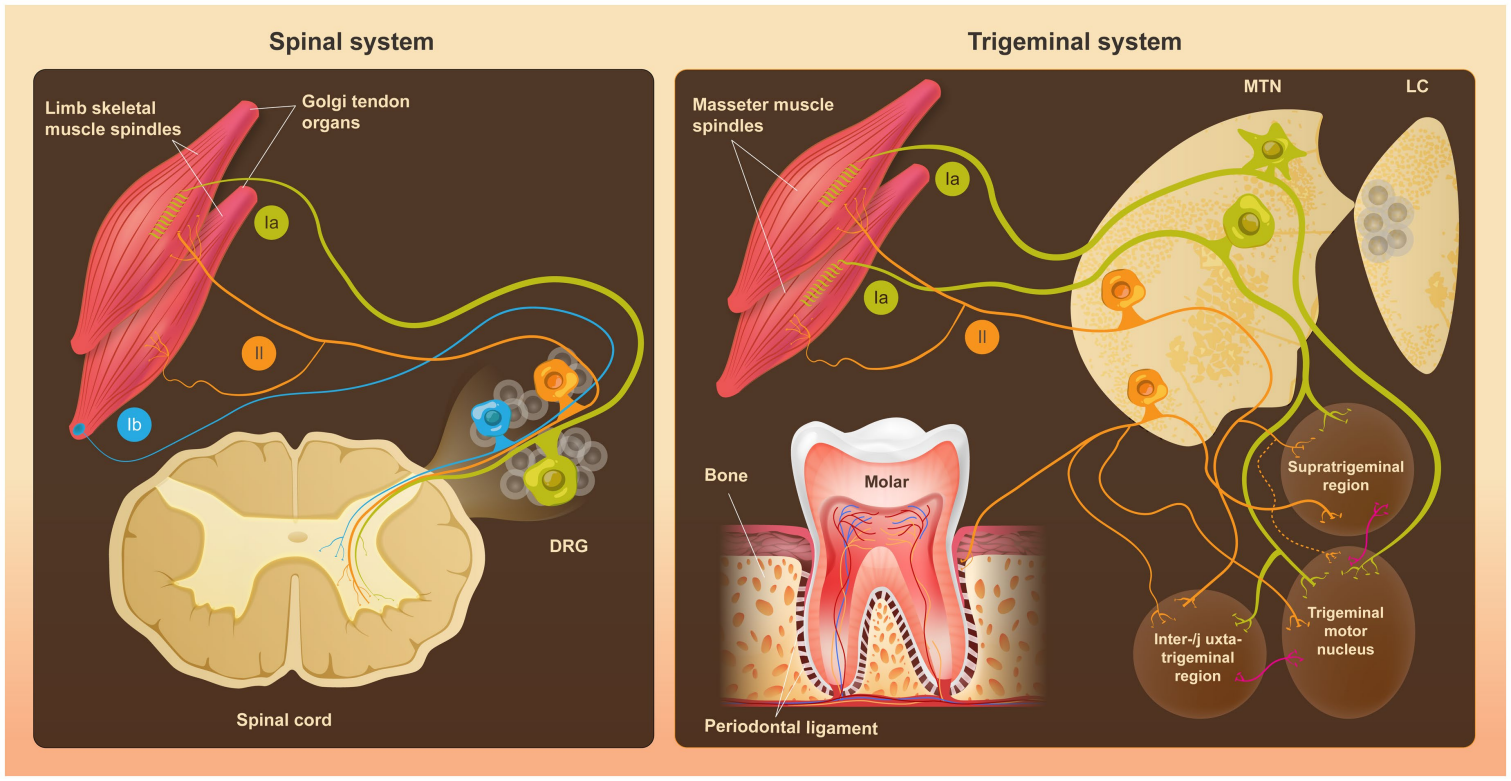
Comparison of anatomical organization and central projections of dorsal root ganglion (DRG) proprioceptors and mesencephalic trigeminal nucleus (MTN) neurons. (Left) In the spinal system, peripheral proprioceptive inputs from limb skeletal muscle spindles and Golgi tendon organs (GTOs) are transmitted by DRG neurons, which exhibit a pseudounipolar morphology. DRG proprioceptive afferents are classically classified into three major groups (Ia, Ib, and II), differing in their peripheral targets and central projections to distinct laminae of the spinal cord. (Right) In the trigeminal system, proprioceptive inputs from jaw-closing muscle spindles, the periodontal ligament and, to a lesser extent, extraocular muscles (not shown), are conveyed by MTN neurons. Unlike DRG neurons, MTN neurons display diverse somatic morphologies (pseudounipolar, bipolar, and multipolar) and form monosynaptic or polysynaptic connections with the trigeminal motor nucleus and premotor areas within the brainstem. MTN neuronal cell bodies are located within the brainstem, in close anatomical proximity to neurons of the locus coeruleus (LC), suggesting coordinated development and potential physiological interactions.

MTN neurons receive proprioceptive input from jaw-closing muscles (Alvarado-Mallart et al., 1975; Nomura and Mizuno, 1985; Shigenaga et al., 1988a), extraocular muscles (Alvarado-Mallart et al., 1975), and the periodontal ligament, which senses mechanical forces at the tooth-bone interface (Nomura and Mizuno, 1985; Shigenaga et al., 1988b) (Figure 1). Axons of MTN neurons predominantly transmit sensory information via monosynaptic connections to the trigeminal motor nucleus (V_mo_), while polysynaptic projections target premotor areas, including the supratrigeminal (V_sup_), intertrigeminal (V_int_), and juxtatrigeminal (V_juxt_) regions; these parallel pathways together support rhythmic jaw movements and broader oromotor control, with preferential routing from distinct peripheral targets (Luo and Li, 1991; Shigenaga et al., 1988a; Shigenaga et al., 1988b) (Figure 1).

It is of particular interest that MTN neurons have been proposed to emerge with jawed vertebrates (gnathostomes), coinciding with the increasing demand for precise regulation of newly acquired oral motor functions such as food breakdown and mastication (Hunter et al., 2001; Lipovsek et al., 2017; Sato, 2021). In extant mammals, modulation of tooth contact and masseter muscle contraction must be tightly coordinated to accommodate variations in ingested food hardness, size, and texture (Hunter et al., 2001; Sato, 2021). Beyond mastication, proprioceptive input from MTN neurons supports complex oral motor behaviors, including swallowing and articulation (Morquette et al., 2012; Sato, 2021). These actions require fast and accurate sensory processing, underscoring the enduring relevance of MTN neurons as a key proprioceptive input for fine-tuned oromotor coordination.

MTN neurons share key morphological and functional attributes with Aα-type DRG proprioceptors, including large cell bodies, thick myelinated axons, and fast conduction properties, consistent with their role in muscle spindle innervation and rapid transmission of stretch-related information (Boyd and Davey, 1968; Hursh, 1939). Classical Aα-type DRG proprioceptors possess large cell bodies, with diameters averaging >50 μm (Le Pichon and Chesler, 2014). Early anatomical descriptions noted apparent similarities between MTN neurons and these peripheral Aα afferents in overall size and morphology (Johnston, 1909). Within the brainstem, MTN neurons are classified as relatively large neurons, with cell body diameters most commonly in the range of approximately 18–30 μm and exceeding 30 μm in a subset of cells (Luo et al., 1991; Turman et al., 1999; Umemura et al., 2010). More recent analyses based on cell body area have further refined these estimates, indicating that MTN neurons rarely exceed ~40 μm in diameter yet constitute one of the largest neuronal populations within the brainstem, a property that renders them morphologically distinctive and readily identifiable (Florez-Paz et al., 2016; Lee et al., 2023).

Despite their overall similarity in cell body size, MTN neurons are distinguished from DRG proprioceptors by a more complex somatodendritic organization. Unlike the predominantly pseudounipolar architecture of DRG neurons, MTN neurons display a spectrum of morphologies, including uni- or pseudounipolar, bipolar, and multipolar forms (Luo et al., 1991). Although pseudounipolar neurons predominate in rodents, multipolar MTN neurons are consistently present as a minor population in mice and rats (up to ~20%) and constitute a substantially larger fraction (approximately 40%) in cats (Luo et al., 1991; Nomura et al., 1985; Umemura et al., 2010). MTN morphology is further shaped by peripheral target identity and postnatal development. Neurons innervating jaw-closing muscles show a higher incidence of multipolar organization, whereas periodontal ligament-projecting neurons are predominantly pseudounipolar and progressively acquire multipolar features after birth (Nomura and Mizuno, 1985; Umemura et al., 2010). In addition, individual MTN neurons extend multiple central axon collaterals to distinct premotor and motor nuclei, including V_sup_, V_int_, and V_juxt_, as well as V_mo_, underscoring their integrative role in oromotor control (Luo et al., 1991; Shigenaga et al., 1988a).

Recent advances in single-cell RNA sequencing (scRNA-seq) have refined the classification of DRG proprioceptor subtypes based on gene expression profiles and developmental trajectories (Sharma et al., 2020; Usoskin et al., 2015; Wu et al., 2021). Building upon these insights, single-cell transcriptomic profiling was recently performed on mouse MTN neurons, revealing their molecular heterogeneity and enabling direct comparisons with canonical DRG proprioceptors (Lee et al., 2023). Notably, the observed transcriptional diversity aligns with the long-recognized morphological heterogeneity of MTN neurons. These findings provide a molecular framework for exploring MTN identity within the broader proprioceptive landscape.

Here, we review current knowledge of the anatomical, molecular, and functional features of MTN neurons, with a particular emphasis on transcriptional properties extending beyond canonical proprioceptor markers defined in DRG neurons (Lee et al., 2023). By comparing MTN neurons with their DRG counterparts, we highlight both conserved and distinctive characteristics that define their specialized proprioceptive identity. We also examine their postnatal specialization in orofacial motor control and outline future research directions to clarify their roles to sensorimotor maturation and developmental disorders.

## 2 Developmental origins shape transcriptional identity

### 2.1 Distinct embryonic origins of MTN neurons and DRG neurons

Most primary somatosensory neurons derive from either the neural crest (NC) or ectodermal placodes; however, the embryonic origin of MTN neurons has long been debated. Early transplantation experiments in avian embryos proposed an NC contribution to MTN neurons, providing important historical perspectives on their developmental origin (Narayanan and Narayanan, 1978). Other interpretations have historically drawn analogies to placode-derived cranial sensory populations; however, direct experimental evidence supporting a placodal contribution to MTN neurons is currently lacking. More recent lineage-tracing and molecular marker studies in amniotes instead localized MTN progenitors to the dorsal midbrain ventricular zone adjacent to the roof plate (Hunter et al., 2001; Lipovsek et al., 2017; Sato, 2021). These findings argue against a migratory NC origin and support a central neuroepithelial source. In contrast, DRG sensory neurons derive exclusively from trunk NC cells that delaminate from the dorsal neural tube and migrate to form bilateral ganglia along the spinal cord (Vermeiren et al., 2020), whereas the trigeminal ganglion (TG) represents an intermediate case with dual contributions from both NC and placodal populations (Erzurumlu et al., 2010). These developmental differences place MTN neurons in a distinct embryological category relative to canonical NC-derived proprioceptors.

Signals from the isthmic organizer, a critical patterning center at the midbrain-hindbrain boundary, play an instructive role in MTN neuron development. Among these, fibroblast growth factor 8 (FGF8) acts as a pivotal inductive cue initiating neurogenesis in the dorsal midbrain (Hunter et al., 2001). MTN neurons are among the earliest-born populations in this region, emerging sequentially from near the isthmus toward more anterior territories (Hunter et al., 2001). Although initial induction can occur independently of sustained isthmic signaling (Lipovsek et al., 2017), experimental manipulations demonstrate that FGF8 is necessary for normal MTN neuron development and can be sufficient to induce ectopic MTN neuron formation. Specifically, ectopic application of FGF8 to anterior or ventral midbrain regions that normally lack MTN neurons is sufficient to induce their formation, whereas FGF8 blockade markedly reduces their number (Hunter et al., 2001). The resulting crescent-shaped distribution of MTN neurons lateral to the periaqueductal gray (PAG) likely reflects both their dorsal origin and the influence of early isthmic patterning (Sato, 2021).

The temporal expression of pan-sensory transcription factors refines the identity of MTN neurons. Key transcription factors and regulatory cues governing the development and maturation of DRG proprioceptors and MTN neurons are summarized in Table 1. Among these, brain-specific homeobox/POU domain protein 3A (Brn3a, POU4F1/*Pou4f1*) is expressed in dorsal midline progenitors during early neurogenesis, whereas ISL LIM homeobox 1 (Islet1, ISL1/*Isl1*) persists into later stages of differentiation (Hunter et al., 2001; Lipovsek et al., 2017; Ter-Avetisyan et al., 2018). In parallel, wingless-related integration site (Wnt) signaling supports early expansion and axonal outgrowth, although it is not required for initial fate specification (Lipovsek et al., 2017). Although primarily associated with temporal aspects of MTN neuron development, the dorsally localized nature of Wnt signaling suggests a potential role in spatial patterning as well (Lipovsek et al., 2017).

**Table 1:** Developmental and maturational factors for DRG proprioceptors and MTN neurons.

Function	System		References
	Dorsal root ganglion (spinal proprioceptors)	Mesencephalic trigeminal nucleus neuron (trigeminal proprioceptors)	
Embryonic origins	Neural crest	Debated (neural crest vs. dorsal midbrain neuroepithelium)	Hunter et al. (2001), Narayanan and Narayanan (1978), and Sato (2021)
Pan-sensory early differentiation	*Pou4f1* (Brn3a), *Isl1* (Islet1)	*Pou4f1* (Brn3a), *Isl1* (Islet1)	Hunter et al. (2001), Lipovsek et al. (2017), and Ter-Avetisyan et al. (2018)
Early sensory neurogenesis	*Neurog1*, *2* (Neurogenin1, 2)	–	Ma et al. (1999) and Vermeiren et al. (2020)
Axonal outgrowth	Wnt signaling	Wnt signaling / FGF8	Hunter et al. (2001) and Lipovsek et al. (2017)
Migratory/trajectory/positioning	No direct counterpart identified	*Onecut1–3* (coordinate positioning with the locus coeruleus)	Espana and Clotman (2012)
Neurotrophic Dependency/survival	NT-3/*Ntrk3* (TrkC)	NT-3 / *Ntrk3* (TrkC), BDNF / *Ntrk2* (TrkB)	Ernfors et al. (1994), Huang and Reichardt (2001), and Matsuo et al. (2000)
Proprioceptor refinement	*Runx3* (*Ntrk2* repression)	Not detected (*Runx3*)	Inoue et al. (2007), Lee et al. (2023), and Levanon et al. (2002)
Axon Bifurcation	No direct counterpart identified	*Npr2* (NPR2)	Ter-Avetisyan et al. (2018)
Muscle-specific targeting	*Etv1* (ER81)	*Etv1* (ER81)*	De Nooij et al. (2013) and Imai and Yoshida (2018)
Matured proprioceptor markers	*Runx3*, *Ntrk3* (TrkC), *Pvalb* (Parvalbumin), *Whrn* (Whirlin), *Lmcd1* (for Ia), *Chad* (for Ib), *Fxyd7* (for II)	*Ntrk3* (TrkC), *Ntrk2* (TrkB)*, *Pvalb* (Parvalbumin)*, *Whrn* (Whirlin)*, *Calb1* (Calbindin D28k)	Lee et al. (2023) and Wu et al. (2021)

Asterisks (*) indicate genes with heterogeneous expression in MTN neurons during late postnatal development (P21–P28). Subtype-specific markers (Groups Ia, Ib, and II) are less clearly segregated in MTN neurons. Brn3a, brain-specific homeobox/POU domain protein 3A; EGR2, early growth response 2; Wnt, wingless-related integration site; FGF8, fibroblast growth factor 8; NT-3, neurotrophin 3; BDNF, brain-derived neurotrophic factor; Trk, tropomyosin receptor kinase; NPR2, natriuretic peptide receptor 2; ER81, ETS variant 1.

Consistent with this, MTN neurons show a distinctive migratory trajectory. After emerging from the dorsal midbrain, they migrate ventrolaterally in close spatiotemporal association with locus coeruleus neurons, reflecting a shared developmental program (Espana and Clotman, 2012). This developmental trajectory requires the Onecut family of transcription factors, including *Onecut1*, *Onecut2*, and *Onecut3*, for proper MTN neuron differentiation and positioning (Espana and Clotman, 2012). In Onecut-deficient mice, both MTN neurons and locus coeruleus neurons are severely reduced or absent, underscoring the essential role of this regulatory axis in their development. Collectively, coordinated transcriptional programs and guided migration define the unique ontogeny of MTN neurons, distinguishing them from peripheral sensory populations such as the DRG.

### 2.2 Divergent transcriptional programs shaping proprioceptive fate

Although MTN neurons and DRG neurons arise from distinct embryonic lineages, both ultimately acquire a proprioceptive phenotype characterized by muscle spindle innervation and stretch-sensitive signaling (Vermeiren et al., 2020). In DRG neurons, sensory identity is established through a well-defined transcriptional hierarchy. Neurogenin 2 (Ngn2, NEUROG2/*Neurog2*), a proneuronal basic helix–loop–helix factor, initiates neurogenesis and activates *Pou4f1* and *Isl1*, which specify pan-sensory neuron identity across cranial and spinal ganglia (Lallemend and Ernfors, 2012; Ma et al., 1999). This core program induces the expression of tropomyosin receptor kinase C (TrkC/*Ntrk3*), a hallmark receptor of myelinated, Aα-type proprioceptors innervating muscle spindles (Le Pichon and Chesler, 2014; Vermeiren et al., 2020). TrkC and its ligand neurotrophin 3 (NT-3) are essential for proprioceptor survival and maturation, as NT-3 knockout models show a profound loss of limb-innervating afferents (Ernfors et al., 1994; Huang and Reichardt, 2001).

This fate is further refined by runt-related transcription factor 3 (*Runx3*), which is activated in immature TrkB/C hybrid neurons during development and is required to establish and maintain a solitary TrkC^+^ proprioceptive phenotype by repressing TrkB and preventing overlap with Aβ mechanoreceptors (Inoue et al., 2007; Levanon et al., 2002; Oliver et al., 2021; Sharma et al., 2020). Loss of *Runx3* results in defective central projections and impaired motor discoordination, highlighting its critical role (Shin et al., 2020). Downstream of TrkC signaling, Ets variant protein ER81/*Etv1* governs peripheral innervation and muscle-specific targeting (de Nooij et al., 2013; Imai and Yoshida, 2018). Mature DRG proprioceptors co-express *Runx3*, TrkC/*Ntrk3*, ER81/*Etv1*, parvalbumin/*Pvalb*, and whirlin/*Whrn*, forming a robust transcriptional signature of spindle afferents (Oliver et al., 2021; Wu et al., 2021). Collectively, this canonical transcriptional cascade establishes a robust proprioceptive identity in DRG neurons.

In contrast, MTN neurons follow a distinct molecular trajectory. Although they predominantly express *Ntrk3* and the calcium-binding protein Calbindin D28k/*Calb1*, a substantial subset also co-expresses *Ntrk2*, and notably, *Runx3* is absent (Lee et al., 2023; Senzaki et al., 2010). Moreover, the expression of proprioceptor marker genes, such as *Etv1*, *Pvalb*, and *Whrn*, is highly heterogeneous among MTN neurons, suggesting partial divergence from the canonical DRG proprioceptive transcriptional program (Lee et al., 2023). The sustained expression of *Ntrk3* in the absence of *Runx3* suggests the existence of an alternative regulatory mechanism independent of *Runx3*, which suggests a possibility that requires further investigation (Lee et al., 2023; Senzaki et al., 2010).

This divergence is further evident in neurotrophic dependencies. Whereas DRG proprioceptors critically depend on NT-3 and TrkC signaling for survival, MTN neurons are only partially affected by the loss of either component. Deletion of NT-3 or TrkC significantly reduces number of MTN neurons but does not eliminate them (Huang and Reichardt, 2001; Matsuo et al., 2000). Further removal of brain-derived neurotrophic factor (BDNF) or its receptor TrkB leads to additional depletion of MTN neurons, suggesting a more distributed trophic support system (Huang and Reichardt, 2001; Matsuo et al., 2000).

Although DRG proprioceptors transiently pass through a TrkB/C hybrid state around embryonic day (E) 10.5–11.5 before segregating under the control of *Runx3* (Lallemend and Ernfors, 2012), it is noteworthy that in MTN neurons, *Ntrk2* expression persists in a subset of cells, as observed through 4 weeks after birth (Lee et al., 2023). Collectively, these findings indicate that, although DRG proprioceptors and MTN neurons converge on a proprioceptive phenotype, they are specified and maintained through distinct transcriptional programs and neurotrophic dependencies. This contrast underscores their divergent developmental origins and specialized signaling environments, illustrating how functional convergence can arise from molecular diversity.

## 3 Unique transcriptional landscape of mature MTN neurons

### 3.1 Muscle spindle-specific proprioceptor signatures

Muscle spindle afferents in the limb have traditionally been divided into Group Ia and II afferents, which terminate on intrafusal fibers within muscle spindles and are distinguished by their preferential sensitivity to dynamic and static components of muscle stretch, respectively (Matthews, 1964). Group Ib afferents, by contrast, innervate Golgi tendon organs (GTOs) and primarily encode muscle force rather than length. Although this classification was originally developed for spinal proprioceptors, anatomical and physiological studies indicate that trigeminal proprioceptors, particularly MTN neurons, conform to a broadly similar functional organization (Jerge, 1963; Shigenaga et al., 1988a). In the cat trigeminal system, MTN afferents with Ia-like properties preferentially terminate in the dorsolateral subdivision of the V_mo_, whereas II-like afferents project mainly to premotor regions such as V_sup_ and V_int_ (Shigenaga et al., 1988a). This laminar projection pattern aligns with a classical lesion study showing that selective ablation of MTN neurons results in degeneration of jaw-closing muscle spindle afferents while sparing GTOs-associated afferents in the same muscles (Jerge, 1963). MTN neurons therefore appear to be predominantly associated with muscle spindle innervation rather than GTO pathways (Figure 1).

Electrophysiological and pharmacological studies further support the functional specificity of MTN afferents. Recordings from these fibers reveal a mixture of dynamic (phasic) and static (tonic) response properties. Afferents with Ia-like characteristics show pronounced sensitivity to stretch velocity, while II-like fibers respond preferentially to sustained muscle stretch (Bewick and Banks, 2015; Matthews, 1964). Pharmacological manipulation exposes additional heterogeneity. Application of succinylcholine to rat masseter afferents differentially alters their dynamic sensitivity, either by strongly enhancing it or by having minimal effect, suggesting afferent subtype heterogeneity (Masri et al., 2006). Unlike the clear separation of primary and secondary endings observed in cats, jaw-muscle afferents in rodents frequently display intermediate response dynamics (Banks et al., 2021; Masri et al., 2006). These patterns suggest that the masseter muscle integrates proprioceptive input from both dynamically sensitive bag_1_ fibers and static-sensitive secondary endings associated with bag_2_ and chain fibers (Masri et al., 2006).

Functional diversity among MTN neurons is reflected at the molecular level. In limb muscles innervated by DRG proprioceptors, acetylcholine receptors are enriched in the equatorial region of intrafusal fibers, where they regulate spindle sensitivity through cholinergic signaling (Gerwin et al., 2019; Zhang et al., 2014). In jaw-muscle proprioceptive circuits, transcriptomic analyses identify subtype-specific expression of acetylcholine receptor genes, providing a molecular correlate for heterogeneous cholinergic modulation observed across these afferents (Lee et al., 2023).

Glutamatergic transmission represents another shared feature of proprioceptive afferents. Vesicular glutamate transporters (vGLUTs) enable activity-dependent glutamate release from sensory endings, contributing to the maintenance of afferent excitability during prolonged muscle stretch (Bewick et al., 2005; Than et al., 2021). Early studies demonstrated robust vGLUT1 expression in muscle spindle terminals, leading to its widespread use as a molecular marker of proprioceptive sensory endings (Bornstein et al., 2023; Woo et al., 2015; Wu et al., 2004). Subsequent single-cell transcriptomic studies further revealed molecular diversity among proprioceptive subclasses, including the co-expression of vGLUT1/*Slc17a7* and vGLUT2/*Slc17a6* transcripts in Group Ia spindle afferents of the DRG (Oliver et al., 2021; Wu et al., 2021).

In MTN neurons, however, vGLUT expression does not follow a uniformly enriched pattern. Classical immunohistochemical studies demonstrated transient vGLUT1 protein expression in neonatal cell bodies, with subsequent restriction to axon terminals in adulthood and no convincing protein-level evidence for vGLUT2 or vGLUT3 (Pang et al., 2006). In contrast, recent single-cell transcriptomic profiling detected mRNA expression of both vGLUT1/*Slc17a7* and vGLUT2/*Slc17a6* in MTN neurons (Lee et al., 2023). Taken together, discrepancies between protein-level localization and transcriptional profiles across developmental stages indicate that vGLUT expression in MTN neurons is context- and stage-dependent, reflecting participation in a broader glutamatergic context rather than a definitive molecular signature of proprioceptive identity on its own.

Integrating molecular markers further refines our understanding of proprioceptor subtype heterogeneity. Recent scRNA-seq studies in DRG proprioceptors identified molecularly distinct Group Ia, Ib, and II populations, characterized by selective expression of genes such as *Lmcd1* for Ia, *Chad* for Ib, and *Fxyd7* for II, along with other transcriptional signatures aligned with functional properties (Wu et al., 2021). When this classification was applied to MTN neurons, two populations resembling Ia- and II-like afferents were identified, whereas Chad-expressing Ib-like neurons were notably absent (Lee et al., 2023).

Other MTN neuronal populations harbored transcriptional profiles distinct from categories defined for DRG neurons, including cells that co-express Group Ia and II markers or entirely lack those genes (Lee et al., 2023). These observations indicate MTN-specific subtypes not encompassed by DRG-based transcriptional classifications. This molecular heterogeneity may correspond to the morphological diversity observed among MTN neurons. For instance, multipolar neurons with smooth dendrites are primarily linked to jaw-closing muscle innervation rather than the periodontal ligament, and may constitute circuit-specialized subtypes potentially suited to dynamic aspects of masticatory control (Luo et al., 1991; Nomura and Mizuno, 1985). Overall, these findings underscore the anatomical and transcriptional specialization of MTN neurons, reflecting adaptation for the precise regulation of oromotor functions.

### 3.2 Ion channel repertoires supporting the proprioceptive function of MTN neurons

Molecular and functional evidence consistently distinguishes MTN neurons from nociceptive trigeminal afferents. Single-cell transcriptomic analyses reveal that nociception-associated ion channels or receptors, including transient receptor potential cation channel subfamily V member 1 (TRPV1) and purinergic receptor P2X_3_, are not detectably expressed in MTN neurons, underscoring a clear molecular segregation from nociceptive TG neurons (Lee et al., 2023). This transcriptomic separation is reinforced by functional data. Electrophysiological recordings show that TRPV1 activation fails to evoke inward currents in MTN neurons, with only minimal inward currents observed following purinergic stimulation (Connor et al., 2005; Kim et al., 2010). Taken together, these observations support a functional segregation of masseter muscle afferents, whereby nociceptive populations project to the TG, whereas MTN neurons constitute a distinct population specialized for proprioceptive muscle spindle signaling.

Neuropeptides classically associated with pain transmission, such as calcitonin gene-related peptide (CGRP) and neuropeptide Y (NPY), are present at very low or undetectable levels in the cell bodies of MTN neurons. Instead, their immunoreactivity is preferentially localized to axons and nerve terminals (Lazarov, 1995). Within the MTN, immunofluorescence is often confined to punctate structures surrounding the cell bodies, a pattern that contrasts sharply with the robust somatic expression of neuropeptides, including CGRP, commonly observed in nociceptive TG neurons (Lazarov, 1995). This spatial segregation further highlights the distinct molecular identity of MTN neurons relative to trigeminal nociceptors.

Importantly, MTN neurons are not defined solely by the absence of nociceptor markers. In contrast to nociceptive TG neurons, they employ mechanosensitive ion channel repertoires tailored to the demands of proprioceptive signaling and precise control of jaw motor function. For example, Piezo2, the principal mechanotransducer in muscle spindle afferents, is robustly expressed in MTN neurons (Florez-Paz et al., 2016) and is likely to serve as the dominant driver of mechanotransduction in jaw-closing muscles, where rapid and high-fidelity feedback is essential. In contrast, ASIC3, an acid-sensing ion channel enriched in limb proprioceptors and implicated in static stretch detection (Lin et al., 2016), exhibits minimal functional sensitivity to extracellular acidification in MTN neurons, as assessed by electrophysiological recordings (Connor et al., 2005). Consistent with this functional profile, transcriptomic analyses report low levels of *Asic3* mRNA in MTN neurons (Lee et al., 2023), further distinguishing them from limb proprioceptive afferents. Accordingly, this selective deployment of mechanosensitive ion channels supports fast proprioceptive signaling and precise regulation of jaw motor output in MTN neurons.

Beyond mechanotransduction, MTN neurons rely on a coordinated repertoire of voltage-gated conductances to support high temporal fidelity in orofacial proprioceptive signaling. TTX-sensitive sodium channel isoforms, including Na_V_1.1, Na_V_1.6, and Na_V_1.7, are expressed in MTN neurons and contribute to rapid depolarization and reliable action potential initiation (Enomoto et al., 2007). While detailed subtype distributions have been characterized most extensively in muscle spindle afferents from limb muscles (Carrasco et al., 2017), these channels are thought to support high-frequency firing and precise spike timing in jaw proprioceptive circuits. Multiple classes of voltage-dependent K^+^ currents further shape excitability and spike timing in MTN neurons. Delayed rectifier and A-type K^+^ currents regulate action potential repolarization and firing adaptation (Del Negro and Chandler, 1997; Hsiao et al., 2009), while Ca^2+^-activated K^+^ conductance dynamically couples intracellular calcium signals to membrane excitability during repetitive firing (Dapino et al., 2023).

In parallel, voltage-gated Ca^2+^ currents contribute to the intrinsic electrophysiological phenotype of MTN neurons by shaping firing patterns and activity-dependent modulation of excitability. Although Ca^2+^ influx is also essential for neurotransmitter release at synapses with trigeminal motoneurons in the V_mo_, the specific calcium channel subtypes mediating synaptic transmission in this circuit remain to be identified. In addition, hyperpolarization-activated cyclic nucleotide-gated (HCN) channels generate I_h_ currents that contribute to firing stability during sustained activity. Early electrophysiological studies provided the first descriptions of HCN-mediated currents in MTN neurons (Khakh and Henderson, 1998), and subsequent work further clarified their roles in excitability control and firing stability (Davoine and Curti, 2019; Kawasaki et al., 2018; Won et al., 2019). Collectively, these conductances help maintain reliable signal transmission during repetitive or prolonged proprioceptive activation in the orofacial system.

In addition to these intrinsic conductances, proprioceptive signaling in MTN neurons is shaped by synaptic mechanisms operating in distinct cellular compartments. At the level of MTN neuron axon terminals forming monosynaptic contacts with trigeminal motoneurons, fast glutamatergic transmission relies predominantly on non-NMDA receptor-dependent mechanisms, consistent with the physiological requirement for rapid and reliable signal transfer in jaw-closing reflex pathways (Chandler, 1989).

At the somatodendritic level of MTN neurons, neuronal excitability and firing dynamics are shaped by the convergence of multiple modulatory inputs. Activation of group I metabotropic glutamate receptors induces resonance-dependent membrane oscillations and can switch MTN neurons from a single-spiking mode to a bursting firing pattern (Chung et al., 2015). Serotonergic modulation provides an additional layer of control, acting through a cAMP–protein kinase A signaling cascade to regulate persistent sodium currents and membrane excitability (Tanaka and Chandler, 2006). In a complementary manner, NT-3 signaling effectively enhances membrane potential oscillations and stabilizes repetitive firing (Yamuy et al., 2000). Dopaminergic boutons have also been identified within the MTN, pointing to further neuromodulatory influences, although their precise functional contribution has yet to be determined (Liem et al., 1997). Rather than acting in isolation, these compartment-specific mechanisms enable MTN neurons to integrate peripherally evoked proprioceptive input with central neuromodulatory signals, thereby supporting temporally precise and adaptable control of orofacial motor behavior.

### 3.3 Transcriptional signatures and circuit specializations beyond canonical proprioceptor markers

In addition to the well-established proprioceptor markers such as *Pvalb* (a calcium-binding protein) and *Whrn* (a scaffolding protein), MTN neurons express a set of transcription factors associated with proprioceptive identity, including Brn3a/*Pou4f1*, Islet1/*Isl1*, ER81/*Etv1*, and members of the Onecut family, along with *Ntrk3* (Lee et al., 2023) (Table 1). These genes have been broadly associated with the development and maintenance of proprioceptor identity. However, MTN neurons diverge from DRG proprioceptors in ways that extend beyond these canonical markers. Comparative transcriptomic analyses reveal distinct expression profiles of ion channels, neurotransmitter receptors, and synaptic proteins, suggesting functional specializations that cannot be fully explained by a shared developmental program. These molecular differences are well positioned to influence conduction properties, central projection patterns, and postsynaptic connectivity, distinguishing MTN neurons from their spinal counterparts.

This transcriptional heterogeneity likely reflects the unique anatomical context and circuit integration of MTN neurons. In addition to forming direct monosynaptic connections with jaw-closing motoneurons, axons of MTN neurons give rise to collateral projections that reach a wide range of premotor and integrative regions, including the V_sup_, V_int_, and V_juxt_ areas, the lateral reticular formation, and the cerebellum (Nomura and Mizuno, 1985; Sato et al., 2025; Shigenaga et al., 1988a). Such distributed connectivity implies that MTN neurons are unlikely to represent a uniform proprioceptive population. Instead, distinct molecular programs may support projection-specific wiring and sensorimotor functions within MTN neurons. How these transcriptionally defined subtypes correspond to anatomical organization and physiological output, however, has yet to be clearly resolved.

A concrete example linking gene expression to circuit organization is provided by the C-type natriuretic peptide (CNP)–natriuretic peptide receptor 2 (NPR2/*Npr2*)–cyclic guanosine monophosphate (cGMP) signaling pathway, which is required for proper development of MTN axons (Ter-Avetisyan et al., 2018). During normal development, MTN axons bifurcate within the hindbrain, and this process critically depends on *Npr2* signaling. Conditional deletion of *Npr2* abolishes axonal bifurcation and leads to a measurable reduction in maximal bite force, establishing a direct link between a specific molecular pathway, circuit wiring, and motor performance. This case illustrates how transcriptional programs in MTN neurons can be translated into circuit-level organization with clear behavioral consequences. An important next step will be to determine how MTN neuron-specific gene expression profiles align with defined projection patterns and anatomical subtypes, and how these transcriptional–anatomical–functional relationships collectively contribute to the coordination of mastication and other fine orofacial motor behaviors.

## 4 Postnatal specialization of MTN neurons for oral sensorimotor control

### 4.1 Behavioral and sensorimotor adaptations during the suckling-to-mastication transition

In early postnatal life, mammals shift from instinctive suckling to voluntary chewing behavior, a transition that typically occurs around the time of weaning. This shift represents a critical milestone in the maturation of oral sensorimotor control (Thiels et al., 1990). Suckling, the primary neonatal feeding strategy, involves rhythmic orofacial movements mainly driven by the lips, tongue, and facial muscles to generate negative intraoral pressure (Westneat and Hall, 1992). By contrast, mastication demands more complex and patterned activation of jaw-closing muscles, coordinated tongue motion, and refined proprioceptive feedback to facilitate the breakdown of solid food (Morquette et al., 2012).

Although both suckling and mastication behaviors are rhythmic, they engage distinct motor pathways and muscle groups. Suckling predominantly recruits jaw-opening muscles, whereas mastication relies on powerful and precisely timed contractions of jaw-closing muscles (Morquette et al., 2012). While MTN neurons innervate both muscle groups, muscle spindles are largely restricted to jaw-closing muscles, and jaw-opening muscles such as the digastric contain few or no spindles, resulting in minimal proprioceptive input (Kidokoro et al., 1968). This asymmetry in spindle distribution places MTN neurons in a position to selectively support the emergence of mastication-specific sensory feedback. Consistent with this interpretation, a classical lesion study demonstrated selective degeneration of MTN afferents projecting to jaw-closing muscles, reinforcing their specialized role in conveying spindle-derived proprioceptive signals required for chewing (Alvarado-Mallart et al., 1975).

In rodents, the behavioral transition unfolds gradually during the second and third postnatal weeks, coinciding with weaning and the introduction of solid food intake (Thiels et al., 1990). Notably, electrophysiological evidence suggests that elements of the masticatory motor program emerge before the overt behavioral shift. Electromyographic recordings detect coordinated chewing-related muscle activity as early as postnatal day (P) 12, indicating that maturation of the underlying motor circuits precedes the visible adoption of mastication (Westneat and Hall, 1992). This sequence appears conserved across mammals, with species-specific differences in timing reflecting variation in neuromuscular developmental rates rather than fundamental differences in circuit organization (Morquette et al., 2012; Narayanan et al., 1971).

Despite this well-characterized behavioral and physiological timeline, the neural mechanisms driving the transition remain incompletely understood. It is unclear whether circuits supporting suckling and mastication are established as independent programs during embryogenesis and recruited sequentially, or whether postnatal remodeling of afferent pathways occurs in response to muscle maturation and changing behavioral demands (Morquette et al., 2012). Resolving this issue places MTN neurons at the center of investigation. In particular, it remains to be determined whether distinct MTN subpopulations undergo molecular, anatomical, or synaptic reorganization during postnatal development to accommodate the increasing reliance on spindle-based feedback. Taken together, these considerations raise the possibility that postnatal adaptation of MTN neurons involves coordinated changes at both the peripheral and central levels, encompassing muscle spindle maturation as well as refinement of brainstem sensorimotor circuits. Dissecting these processes will be essential for understanding how stable yet flexible oral motor behaviors emerge during development.

### 4.2 Peripheral adaptations supporting the transition to mastication

To better understand the proprioceptive role of MTN neurons during this transition from suckling to mastication, it is necessary to consider how the peripheral musculoskeletal and dental structures mature in parallel. This transition entails coordinated remodeling of the peripheral oral system, encompassing sensory end organs, muscles, teeth, and joints. Among these components, the postnatal development of jaw-closing muscle spindles represents a particularly critical adaptation. These proprioceptive end organs are specified prenatally but undergo substantial postnatal growth, including increases in intrafusal fiber number and fiber diameter (Bornstein et al., 2023; Maeda et al., 1988; Sato et al., 2007).

Recent multi-omics analyses have delineated the postnatal maturation of jaw-closing muscle spindles, revealing progressive increases in capsule complexity and dynamic transcriptomic and proteomic remodeling between P3 and P25 (Bornstein et al., 2023). These changes include the emergence of novel molecular markers across multiple spindle compartments, spanning intrafusal fibers, capsule cells, and extracellular matrix components. Notably, capsule cell maturation follows a spatiotemporal gradient from the spindle center toward the poles. Such structural and molecular refinements are likely to tune spindle sensitivity to muscle stretch, thereby providing the structural foundation for precise proprioceptive feedback during mastication. In line with this interpretation, the functional properties of masseter muscle spindles mature substantially over the same period, supporting high-force, repetitive jaw-closing contractions by the end of weaning, in contrast to the low-force, reflexive movements characteristic of suckling. This increase in neuromuscular precision is essential for the safe and efficient processing of solid food.

Peripheral adaptations that support mastication are not restricted to the maturation of muscle spindles. Molar eruption occurs later, between P17 and P20 (Denes et al., 2018; Sunariani et al., 2019; Westneat and Hall, 1992). This event is critical for effective grinding of solid food and coincides with the onset of rhythmic chewing and solid food intake. The tight temporal alignment of dental eruption, muscle maturation, and behavioral change suggests the existence of a developmentally regulated window during which peripheral and neural systems converge to permit functional mastication.

Chewing itself further drives structural remodeling of the masticatory apparatus. Increased masticatory loading induces adaptive changes in the temporomandibular joint and mandible, enhancing jaw mobility and mechanical stability (Buvinic et al., 2020; Kato et al., 2015). Jaw-closing muscles increase in cross-sectional area, while ongoing craniofacial growth improves mechanical leverage for bite force generation (Bornstein et al., 2023; Hurov et al., 1988). Collectively, these postnatal biomechanical and structural transformations not only support efficient, forceful, and repetitive oral motor behaviors but also reshape the proprioceptive demands placed on MTN neurons by altering the magnitude, timing, and complexity of sensory feedback generated during mastication.

### 4.3 Central refinement of MTN neuron circuits during weaning

During the weaning period, the central circuits formed by MTN neurons undergo refinement to support the increasing complexity of masticatory behaviors. Early developmental studies revealed temporally coordinated changes in excitatory glutamatergic signaling within MTN neurons and adjacent premotor regions. Turman and colleagues identified shifts in the expression of ionotropic glutamate receptor subunits, including NMDA (NR1, NR2A/B) and AMPA receptors (GluRs) (Turman et al., 1999; Turman et al., 2000), as well as metabotropic glutamate receptors (mGluRs), including mGluRs 1 and 5 (group I) and mGluRs 2 and 3 (group II), which modulate neuronal excitability and synaptic integration over longer timescales (Turman et al., 2001). These studies indicate that both fast ionotropic and modulatory metabotropic glutamatergic pathways mature in a coordinated manner, refining excitatory signaling within MTN neuron circuits and their associated premotor networks.

For instance, NR1 is broadly expressed from birth, but NR2A/B emerges in a caudal-to-rostral gradient, appearing by P3 in MTN neurons located in the caudal and middle regions, and only by P8 in the rostral region (Turman et al., 1999). This temporospatial expression pattern of NR2A/B may correspond to the differential timing of sensory input from target structures such as the periodontal ligament, which receives earlier innervation than the masseter. Similarly, AMPA receptor expression is not uniformly distributed; some neurons lack detectable AMPA labeling, and subunit-specific profiles vary across regions (Turman et al., 2000). These patterns suggest that AMPA-mediated transmission is selectively refined across MTN subpopulations, contributing to improved synaptic fidelity and temporal precision as chewing behavior emerges.

Building on this foundation, recent scRNA-seq analyses have provided higher-resolution insights into the postnatal maturation of MTN neurons (Lee et al., 2023). By P21, MTN neurons exhibit molecular heterogeneity, with robust expression of *Ntrk3*, the canonical proprioceptive receptor, and a subset retaining relatively high levels of *Ntrk2*. By P28, *Ntrk2* expression declines, though not completely extinguished, resulting in a more uniform *Ntrk3*-dominant profile. These findings indicate that MTN neurons do not appear to acquire *Ntrk3 de novo*, but instead refine an already present proprioceptive identity through the downregulation of developmental transcripts such as *Ntrk2*. Importantly, the refinement of *Ntrk2*/*Ntrk3* expression is not an isolated event but part of a broader molecular transition.

At the transcriptome-wide level, single-cell analyses reveal pronounced developmental shifts in MTN neurons across the weaning period. Genes associated with nervous system development, synapse assembly and organization, and programmed cell death are more highly enriched at P21, whereas genes related to protein synthesis and synapse-associated translational machinery are preferentially enriched at P28 (Lee et al., 2023). These coordinated transcriptional changes are consistent with a shift away from large-scale circuit construction toward finer synaptic refinement, accompanied by increased reliance on localized protein synthesis. In line with this interpretation, postnatal maturation of proprioceptive circuits is increasingly understood to involve activity- and target-dependent refinement of central synapses. In the spinal cord, for example, Group Ia proprioceptive afferents undergo selective stabilization and pruning of their central projections well after birth, following initial circuit assembly (Arber et al., 2000). The molecular transition observed in MTN neurons during weaning likely reflects ongoing refinement of oral proprioceptive circuits associated with increasing demands for precise jaw motor control.

These transcriptional changes are further complemented by morphological observations, which reveal postnatal structural remodeling of MTN neurons. Specifically, a previous study reported that MTN neurons innervating the masseter muscle undergo minimal postnatal morphological alterations, whereas those projecting to the periodontal ligament show a significant reduction in cell body diameter between P7 and P14. In parallel, the periodontal ligament-innervating population progressively adopts a multipolar phenotype, with its initial emergence at P14 aligning with tooth eruption in rats and further consolidation by P70 (Umemura et al., 2010). Consistent with these observations, more recent work documented a pronounced decrease in the cell body size of MTN neurons between P21 and P28 (Lee et al., 2023), providing further support for progressive morphological consolidation during circuit maturation. Collectively, these findings suggest that structural maturation of MTN neurons is shaped by their peripheral targets, reflecting region-specific postnatal remodeling within the nucleus.

At the circuit level, MTN neurons refine their connectivity in parallel with these molecular and morphological changes. By the weaning stage, they form robust monosynaptic connections onto jaw-closing motoneurons in the V_mo_, providing the anatomical substrate for the masseteric stretch reflex (Luo and Li, 1991; Nomura and Mizuno, 1985; Nomura et al., 1985). This highly specific wiring ensures rapid, high-fidelity proprioceptive feedback for precise control of bite force and jaw position. These multi-level refinements—molecular, morphological, and synaptic—enable MTN neurons to transition from immature, heterogeneous states into a specialized and efficient circuit optimized for mastication.

## 5 Conclusion

MTN neurons possess a distinct molecular, anatomical, and functional identity tailored to their role as primary proprioceptors for jaw-closing muscles and the periodontal ligament. Like DRG proprioceptors, MTN neurons distribute proprioceptive signals through branching central projections to multiple targets. Unlike DRG neurons, however, their cell bodies are embedded within the brainstem, where their developmental trajectory and signaling properties are shaped by close interactions with neighboring neuronal populations, including the locus coeruleus. At the same time, MTN neurons form monosynaptic connections with trigeminal motoneurons, constituting the dominant circuit for high-speed proprioceptive feedback required for coordinated mastication. Their postnatal maturation further involves tightly orchestrated transcriptional, morphological, and synaptic refinements, resulting in a proprioceptive system precisely tuned to the biomechanical demands of mastication. Taken together, these features make MTN neurons a powerful model for understanding how behaviorally relevant proprioceptive circuits mature.

Animal studies provide additional evidence that masticatory activity itself modulates oral neuromuscular development. For example, a soft or liquid diet during postnatal stages reduces bone formation and delays the maturation of both masseter muscle spindles and temporomandibular joint cartilage (Kato et al., 2015). In parallel, research on masseter spindles demonstrates a protracted postnatal maturation process, with differentiation beginning late in gestation, continuing beyond weaning, and followed by an age-related decline in adulthood (Maeda et al., 1988). Although these observations document measurable changes in spindle maturation, whether dietary conditions actively reshape spindle developmental programs remains unresolved and warrants further investigation. Taken together, these findings indicate that intrinsic developmental programs and environmental inputs converge to shape the maturation of masticatory proprioceptors, with direct relevance for MTN neuron refinement and the emergence of coordinated oral motor control.

Accumulating evidence also indicates that MTN neuron-associated circuits may be particularly sensitive to early-life perturbations in feeding experience. In rodents, early weaning induces long-lasting alterations in ingestive behavior and hypothalamic circuit organization (Alves et al., 2025). In humans, shortened breastfeeding duration has been associated with increased risk of neurodevelopmental disorders, including attention-deficit/hyperactivity disorder (ADHD), autism spectrum disorder (ASD), and schizophrenia, whereas prolonged breastfeeding correlates with improved cognitive outcomes. These associations do not imply a direct causal role for MTN neurons but underscore the sensitivity of early sensorimotor circuits to feeding-related experience, highlighting the early postnatal period as a critical window for circuit maturation.

Despite their central role in mastication, MTN neurons remain underexplored in the context of developmental disorders and motor dysfunction. Given their position at the sensory-motor interface, disruption of MTN neuron circuits could plausibly contribute to pediatric feeding difficulties, bruxism, and craniofacial pain syndromes (Masri et al., 2005; Ono et al., 2010). Beyond motor control, growing evidence links masticatory dysfunction to cognitive decline and Alzheimer-like pathology. Both animal models and human studies report associations between impaired mastication and structural or functional changes in the basal forebrain, hippocampus, and cortex (Kang et al., 2024), and reduced proprioceptive input has been correlated with β-amyloid accumulation (Yesantharao et al., 2023). These observations raise the possibility that altered MTN neuron-mediated proprioceptive signaling, which is sensitive to early-life mechanical and nutritional environments, may influence or reflect broader neurodegenerative processes with consequences for long-term cognitive health.

MTN neurons thus emerge as a unique nexus where developmental programming, environmental experience, and synaptic precision converge. Their anatomical accessibility and functional specificity make them an exceptional system for dissecting how proprioceptive circuits develop, adapt, and malfunction across the lifespan. Progress in this field will depend on integrative strategies that combine stage-resolved single-cell and multi-omics analyses with lineage tracing, trajectory inference, circuit-level mapping, and behavioral phenotyping, enabling systematic dissection of how MTN neuronal diversity arises, how subtypes specialize, and how they contribute to oral sensorimotor control. Such approaches will be essential for identifying windows of vulnerability and plasticity, with clear translational implications for developmental disorders, neuromuscular dysfunction, and neurorehabilitation.
